# Total laparoscopic distal pancreatectomy for a benign appearing tumor: a case report

**DOI:** 10.4076/1757-1626-2-8468

**Published:** 2009-07-22

**Authors:** Christopher Kosmidis, Christopher Efthimiadis, George Anthimidis, Marios Grigoriou, Evangelos Toulis, Sofia Levva, Ioannis Prousalidis, Epaminondas Fachantidis

**Affiliations:** 1 Department of Surgery, Interbalkan European Medical CenterAsklipiou 10, 57 001 Pylaia, ThessalonikiGreece; 2 Propedeutic Surgical Department, AHEPA University Hospital, Aristotle’s University of ThessalonikiSt. Kiriakidi 1, 546 36 ThessalonikiGreece; 3 Department of Internal Medicine, Interbalkan European Medical CenterAsklipiou 10, 570 01 Pylaia, ThessalonikiGreece

## Abstract

**Introduction:**

Therapeutic laparoscopy of the pancreas is still described as experimental surgery by many surgeons. Many issues remain to be clarified in determining the future of this new method.

**Case presentation:**

The objective of the present study was to present a case of a patient who underwent totally laparoscopic distal pancreatectomy for a benign appearing tumor in the tail of the pancreas and to critically discuss the treatment of the pancreatic remnant and the need to perform splenectomy with or without ligation of the splenic vessels.

**Conclusion:**

Laparoscopic distal pancreatectomy is usually performed en-bloc along with resection of the spleen, for technical reasons, making the operation short and easy. However, it should only be performed in centers with expertise in both pancreatic surgery and advanced laparoscopy. Furthermore, the use of linear stapler to cut the pancreas (4.5-mm staples) seems to prevent fistula formation and ischemia of the pancreatic stump.

## Introduction

Laparoscopic pancreatic surgery is rapidly developing in relation to technological improvements and increasing experience among advanced laparoscopic surgeons. As experience is gained, laparoscopic pancreatic resections may prove to be associated with real advantages during the postoperative period. In recent years, laparoscopic distal pancreatectomy has become an increasingly used technique in the surgical treatment of several pancreatic diseases, including non-malignant tumors [1-3]. However, case selection is crucial and should be based on pathology, clinical features and past medical history. We present herein a case of a patient who underwent total laparoscopic distal pancreatectomy for a benign appearing tumor in the tail of the pancreas.

## Case presentation

**Figure 1.**
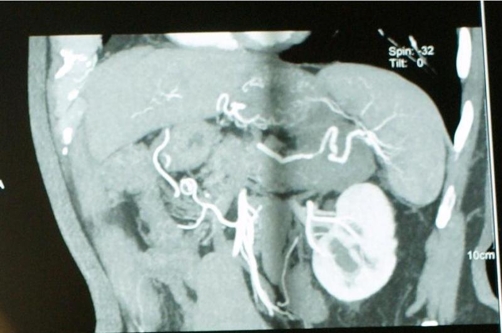
Computed tomography-angiography showing that the tumor distorted and compressed the splenic vessels.

A 72-year-old Greek male patient, which had undergone transurethral resection of prostate 14 years ago, was followed up with contrast enhanced computed tomography (CECT), which revealed a 4.5 × 4 cm macrocystic lesion in the tail of the pancreas. The patient complained for vague postprandial abdominal pain. Physical examination was not contributory. Ultrasonography (US) revealed a 4.5 × 4 cm hypoechoic lesion in the tail of the pancreas as well as cholelithiasis. Magnetic resonance imaging (MRI) also revealed a 4.5 × 4 cm macrocystic lesion in the tail of the pancreas. In addition, computed tomography-angiography (CTA) showed that the tumor distorted and compressed the splenic vessels (Figure 1). The level of serum CEA and CA19-9 were within the normal range. Preoperative evaluation led to the possible diagnosis of mucinous cystic neoplasm of the pancreas. The patient was subjected to laparoscopy. Intraoperatively a cystic mass of approximately 4.5 cm in diameter was found in the tail of the pancreas in addition to atrophy of the adjacent parenchyma. Laparoscopic cholecystectomy and distal splenopancreatectomy was performed. Postoperative recovery of the patient was uneventful, while the drainage was removed and the patient stepped out on the second and fourth postoperative day respectively.

Despite the preoperative diagnosis of mucinous cystadenoma the histology was consistent with a pancreatic pseudocyst, which was subsequenced to moderately differentiated ductal adenocarcinoma. Resection margins were negative, as well as perineural invasion was not present and no metastasis was found in eight peripancreatic resected lymph nodes.

Patient underwent adjuvant chemotherapy, with gemcitabine 1000 mg/m^2^ weekly for 3 weeks followed by a rest week, and for 6 cycles. He tolerated treatment well without side effects. The patient was followed up with US, abdominal CECT and chest X-ray after 6 months. The level of CA19-9 was determined one month after surgery and then every 3 months and it was within the normal range. The patient remains asymptomatic 6 months after the operation.

### Technique

**Figure 2.**
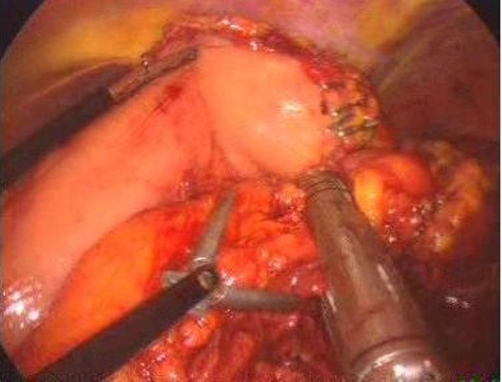
The linear cutter stapling device (Endoscopic Vascular Stapler 45 mm) dissecting the pancreas at the selected resection line at the middle of body.

**Figure 3.**
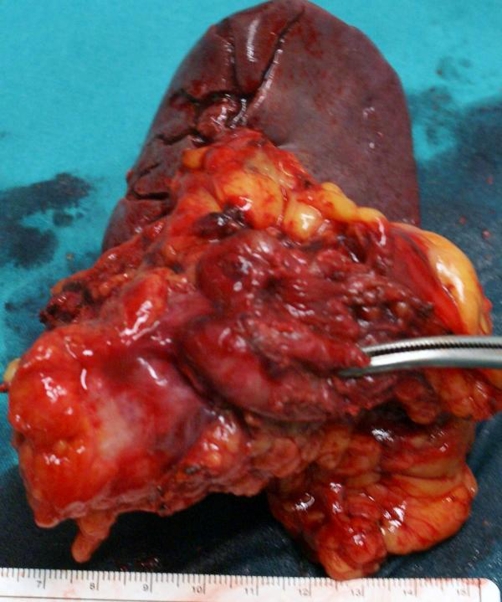
The specimen of the distal pancreas together with the spleen.

The patient was placed in supine position with his legs apart. A gastric tube and a bladder catheter were inserted. The monitors were placed over the left and right shoulders of the patient. A Veress needle was inserted under the umbilicus to induce pneumoperitoneum. The intra-abdominal pressure was monitored and maintained at around 12 mmHg. A 10 mm camera port was inserted in the umbilical position. A 10 mm port was placed in the subxiphoid area. Another two 5 mm trocars were placed, one near the midclavicular line (in the right subcostal area), and another on the anterior axillar line. The operating table allowed changing the patient's position easily: a slight (35°) anti-Trendelenburg tilt was obtained. We used a 10 mm 30-degree telescope for visualization. Laparoscopic cholecystectomy was initially performed. Subsequently the operating table was rotated about 30° to the right. The surgeon stood between the legs of the patient, while the first and second assistants, respectively, stood on the left and right of the operating surgeon. The scrub nurse was on the right side of the operating surgeon. A 12 mm port, through which the stapler could be introduced, was inserted in the left upper quadrant. The operating surgeon used the instruments placed in the right subcostal area and in the left side. The pancreas was explored through the infragastric access. Using an atraumatic grasper introduced through the subxiphoid trocar, the assistant grasped the stomach at the great curvature and raised it. The operating surgeon used an ultrasound dissector to mobilize the splenic flexure. All suspensory ligaments of the spleen were divided, including the short gastric vessels. The gastrocolic omentum was divided, preserving the gastroepiploic artery. Thus, a window was opened in the gastrocolic ligament, below the gastroepiploic arch. The lesser sac was entered and the window was then enlarged to expose the pancreas. The peritoneum overlying the inferior border of the pancreas was incised, and via this largely avascular plane of dissection the lower border of the pancreas was elevated, allowing for retroperitoneal pancreatic access. A linear cutter stapling device [ENDOPATH®, ETS Flex 45 Endoscopic Articulating Linear 45 mm staple line, 2.5 mm Staple Leg Length (Vascular/Thin) 45 mm Vascular/Thin, Johnson & Johnson, USA] was then placed across the pancreas at the selected resection line (the middle of body). The splenic vein and artery were deeply embedded within the parenchyma of the pancreas. The splenic vessels were dissected en bloc together with the parenchyma, using 2 mechanical stapler fires (Figure 2). Additionally, an endoscopic clip was applied to the splenic artery at the level of transection of the pancreas. Once the pancreatic remnant was dissected, the spleen was mobilized by sectioning any remaining attachments. The specimen was removed using a gel-port through an incision in the left upper quadrant (Figure 3). A Jackson-Pratt drain was placed in the bed of the pancreatic dissection and drawn out through the 5-mm port site on the anterior axillar line. It was removed on the second day postoperatively, once it was determined that there was no pancreatic fluid leak.

## Discussion

Although more than a few laparoscopic pancreatic procedures have been described, debate goes on over which procedures can be safely and effectively performed and which clearly benefit the patient when performed laparoscopically. Laparoscopic distal pancreatic resection has increasingly been described as a feasible and safe procedure.

The most common histotypes of resectable distal pancreatic tumors are currently cystic and endocrine neoplasms, which are frequently benign and usually diagnosed incidentally during ultrasound examinations [3]. In general, the indications for laparoscopic distal pancreatectomy are neuroendocrine tumors and benign appearing tumours [4]. However, Melotti et al reported that laparoscopic resection of ductal carcinomas was safe and oncologically correct [3]. In our case, the preoperative diagnosis was a benign appearing tumor (mucinous cystic neoplasm). However, the histology revealed a pancreatic pseudocyst and a separate area of moderately differentiated ductal adenocarcinoma. The resection margins and the eight removed lymph nodes were disease-free.

Laparoscopic distal pancreatectomy offers patient benefits in terms of postoperative recovery and length of stay, together with acceptable perioperative morbidity and complication rates [5,6]. However, laparoscopic distal pancreatectomy raises two problems: sparing the spleen with or without ligation of the splenic vessels, and treating the pancreatic remnant. Distal pancreatectomy with en-bloc splenectomy has been considered the standard technique for treatment of benign and malignant pancreatic disorders [7-10]. However, splenic preservation has lately been advocated [2,5,7-14]. In patients with malignant tumors in the body-tail of the pancreas, splenectomy has a negative influence on long-term survival after resection. The incidence of diabetes after spleen-preserving distal pancreatectomy for chronic pancreatitis is less than after en-bloc splenectomy. Spleen salvage eliminates the risk of overwhelming infections [4]. The question is whether it should be performed with or without splenic vessel preservation. Kimura et al have described the technique of preserving both the splenic artery and vein [15]. Warshaw has described a technique of distal pancreatectomy in which splenic vessels are ligated both at the level of transection of the pancreas and again at the splenic hilum, leaving the spleen to survive on blood flow through the short gastric vessels [16]. To achieve laparoscopic access and sufficient exposure of the distal pancreas, a number of the short gastric vessels must be divided which, together with a divided splenic pedicle, would likely render the spleen nonviable. Splenomegaly is a contraindication for Warshaw’s method because the increased mass is not sufficiently nourished by the short gastric vessels. There is no doubt that by preserving the splenic artery and vein, the blood supply to the spleen is well maintained and the risk of splenic necrosis and abscess formation is reduced. On the other hand, distal pancreatectomy with conservation of the splenic artery and vein is both time and labour consuming. Dissecting the splenic vessels from the pancreas may be difficult in the presence of tumours distorting and compressing the course of the vessels [4,15,17].

However, in general, distal pancreatectomy is performed en-bloc along with resection of the spleen. The en-bloc pancreatic-spleen resection is usually performed for technical reasons; it makes the operation short and easy [4].

In our case, the spleen was resected en bloc with the distal pancreas because the splenic vein and artery were deeply embedded within the parenchyma of the pancreas.

As in open resections, the major challenge is still the management of the pancreatic stump. The formation of fistulae is a result of the soft tissue typical of the residual pancreas of patients suffering from cystic or endocrine benign tumors [3]. Once the stapler is fired, the surgeon may choose to oversew the staple line as an additional measure to ensure pancreatic ductal closure. Park et al do so routinely [5]. A bipolar vessel sealing device used to divide the pancreas has been described by Matsumoto [18]. According to Melotti et al the best technique to cut the pancreas is using the linear stapler. During their initial experience they used 3.5-mm staples before changing to 4.5-mm staples to prevent ischemia of the pancreatic stump and reduce fistula formation [3]. In our case, we used 4.5-mm staples, in line with the most current results of Melotti et al [3].

Despite extensive preoperative and even intraoperative investigation (including intraoperative ultrasound), there is no minimally invasive substitute for manual palpation and exploration of the pancreas. Recent advances in hand-assisted laparoscopic surgery may address this limitation of totally laparoscopic surgery. The disadvantage of hand-assisted laparoscopic surgery is that often a larger than expected incision for the surgeon’s hand is required; this carries with it the risk of wound infection and incisional hernia, in addition to the obvious functional and cosmetic impairment [5].

## Conclusions

In conclusion, laparoscopic pancreatic surgery is becoming gradually more popular among surgeons. In general, distal pancreatectomy is performed en-bloc along with resection of the spleen for technical reasons, making the operation short and easy. The use of linear stapler to cut the pancreas (4.5-mm staples) seems to prevent fistula formation and ischemia of the pancreatic stump. Our case confirmed the safety and feasibility of laparoscopic distal pancreatectomy in the treatment of pancreatic tumors. It is not possible, however, to draw from this case report any definitive conclusions. Laparoscopic distal pancreatectomy should only be performed in centers with expertise in both pancreatic surgery and advanced laparoscopy. Laparoscopic technology is in a rapid state of development and further improvements might play an important role for the future diffusion of laparoscopic pancreatectomy.

## Abbreviations

CECT: contrast enhanced computed tomography
CTA: computed tomography-angiography
MRI: Magnetic resonance imaging
US: Ultrasonography
